# Gender differences in the relationship between adult attachment and self-identity: A network analysis research among Chinese college students

**DOI:** 10.1371/journal.pone.0351504

**Published:** 2026-06-12

**Authors:** Cui Lyu, Lili Xu, Ying Qian, Jia-Ming Wei

**Affiliations:** 1 ‌‌Department of Students’ Affair, Huaibei Normal University, Huaibei, Anhui, China; 2 College of Education, Huaibei Normal University, Huaibei, Anhui,‌‌ China; Tung Wah College, HONG KONG

## Abstract

**Objective:**

This study aims to investigate the relationship between adult attachment and self-identity among Chinese college students using network analysis, with a specific focus on examining gender differences in network structure, global strength. and edge strength.

**Methods:**

A convenience sampling method was employed, and a total of 624 university students from China were surveyed using the Experiences in Close Relationships Inventory and the Self-Identity Questionnaire developed by Chinese scholars. Network analysis was conducted to estimate the structure of adult attachment and self-identity, identify central and bridge symptoms, and examine gender differences in network structure, global strength, and edge strength.

**Results:**

The self-identity items formed a tightly connected cluster. Temporal disintegration constituted the core node bridging adult attachment and self-identity networks, followed by identity diffusion. Attachment anxiety was the strongest bridge node connecting adult attachment to self-identity, primarily associating with temporal disintegration. Network invariance test indicated no significant gender differences. Global strength invariance test was significantly higher in females than in males. Edge strength invariance test revealed that there were significant differences in the strength of some edges between males and females.

**Conclusion:**

In the adult attachment and self-identity network, temporal disintegration and identity diffusion serve as core nodes, around which close clusters form**.** Attachment anxiety serving as the key bridge. Although the network structure is similar across genders, females show significantly stronger overall network connectivity. These findings highlight the importance of examining both local and global metrics when studying group differences in psychological networks.

## Introduction

Self-identity refers to an individual’s subjective sense of continuity across past, present, and future selves, thereby addressing the fundamental question “Who am I?” both explicitly and implicitly [1]. It also reflects the integration and adaptation of the self within specific environmental contexts [2]. The formation of self-identity involves recognizing one’s position in society and perceiving oneself as an integral part of the social structure, which in turn influences decision-making in domains such as occupation, marriage, and religious beliefs [3]. Erikson identified adolescence and emerging adulthood—particularly the university years—as a critical period for identity development, marking a pivotal transition from adolescence to adulthood [4]. Failure to effectively integrate personal experiences with social roles during this stage may lead to an identity crisis, increasing the risk of academic burnout, interpersonal difficulties, and mental health problems [5].

Self-identity formation is shaped by both internal and external factors. According to Erikson, internal factors stem from the outcomes of lifelong psychosocial developmental stages. Exploration and integration during adolescence and young adulthood involve mobilizing and reorganizing these early internal resources, including personality dispositions, value systems, and imaginings of “what I might become,” which serve as the raw materials and driving forces for identity exploration [4,6]. External factors encompass the sociocultural environment—primarily family and parenting styles, peer groups, school education, and broader sociocultural norms [7]. In the Chinese cultural context, personality traits [8] and parenting styles are particularly salient among internal and external influences, respectively. The continuous interplay between these factors jointly shapes self-identity [4,9]. McLean and Syed further emphasize that self-identity emerges through the dynamic interaction between the individual and culture, evolving within relational contexts [10]. Thus, identity development depends not only on introspection about “who I am,” but also on repeated experiences of how others respond to the self.

Attachment theory provides an alternative perspective for understanding the development of self-identity. Bowlby [11] proposed that the early relationships quality critically shapes how individuals perceive themselves and the world. Early experiences are integrated into cognitive templates of the self and others, known as internal working models, which constitute stable mental representations. These representations not only regulate expectations and behaviors in close relationships but also influence one’s self-positioning and capacity for commitment in social roles [12,13] Different cognitive schemas give rise to distinct types of internal working models, which in turn manifest as different attachment styles [14]. As individuals mature, early attachment relationships are gradually transferred to friends or partners, forming what is known as adult attachment [15,16]. Researchers conceptualize adult attachment along two continuous dimensions: attachment anxiety and attachment avoidance [17,18]. Individuals high in attachment anxiety tend to employ hyperactivating strategies, characterized by a negative self-image, fear of rejection, abandonment anxiety, and excessive self-doubt. Those high in attachment avoidance typically use deactivating strategies, manifesting as discomfort with emotional intimacy, reluctance to depend on others, a preference for self-reliance, and negative perceptions of others and distrust in relationships. When perceiving threat, highly avoidant individuals often adopt maladaptive strategies—such as denying attachment needs, avoiding reliance on others, and distancing themselves from negative emotions or thoughts that may activate the attachment system [19–21]. Early attachment experiences shape the formation of internal working models regarding the self (e.g., “Am I worthy of love and attention?”) and others (e.g., “Are others reliable and accessible?”) [22]. These models profoundly influence the starting point and manner in which individuals explore the question “Who am I?” in adulthood. Cross-sectional studies have found a positive correlation between attachment avoidance and identity diffusion [23,24], and between attachment anxiety and identity foreclosure [25]. However, most of these studies adopt a variable-centered approach, which offers limited insight into the dynamic relationships among specific symptoms or dimensions.

### Research on gender differences in adult attachment

Classic attachment theory was originally formulated on a gender-neutral basis. However, growing evidence indicates that adult attachment is closely associated with social relationships such as romantic partnerships, marriage, and parenting—domains in which gender often plays a significant role in shaping relational dynamics [26]. It is thus plausible that systematic gender differences may also exist in adult attachment itself. Meta-analytic findings have consistently shown that men tend to score higher on attachment avoidance, whereas women report greater levels of attachment anxiety—a pattern that demonstrates cross-cultural consistency [27]. Nevertheless, this general trend has not been fully supported among Chinese university students. Some studies indicate that Chinese women do not necessarily exhibit significantly higher attachment anxiety—a phenomenon potentially attributable to traditional East Asian cultural norms that may suppress emotional expression in women [28]. Conversely, other research has reported that Chinese women scored higher than men on attachment avoidance [29]. A meta-analysis covering data from Chinese college students between 2003 and 2015 concluded that there were no significant gender differences in attachment styles within this population [30]. These inconsistent findings highlight the possibility that sociocultural changes—including rapid economic transformation, evolving family ideologies, and significant improvements in women’s educational attainment and social status—may be contributing to a restructuring of traditional gender roles and enhancing female autonomy in contemporary China. Consequently, earlier conclusions regarding gender differences in adult attachment warrant re-examination within the current sociocultural context.

### Research on gender differences in self-identity

Self-identity formation is inherently embedded in sociocultural contexts [31]. In collectivist cultures like China, self-definition tends to emphasize relational harmony, family obligations, and social role responsibilities [32], whereas Western contexts prioritize individual autonomy and clear interpersonal boundaries [33]. Consequently, Western-derived identity frameworks may inadequately capture the nuances of identity development among Chinese adolescents—particularly commitments driven by familial expectations or explorations situated within relational contexts. To better assess self-identity in this cultural setting, the present study employs a scale developed by Chinese scholars Zheng and Huang [34]. Within Marcia’s identity status model, Chinese samples show distinct gender patterns: males are more likely to exhibit identity foreclosure, while females are more frequently associated with identity diffusion [35]. These findings suggest that the psychological architecture linking adult attachment and identity may differ by gender. However, existing research has not clarified how gender moderates the associations between specific attachment dimensions and identity subcomponents—such as self-consciousness, lack of focus, and identity diffusion.

### The current study

Although prior studies have examined the relationship between adult attachment and self-identity using variable-centered or latent class perspectives [23,25], the two constructs have often been investigated independently: attachment research primarily focuses on the quality of intimate relationships, while identity studies emphasize developmental trajectories and their impact on mental health. Nonetheless, both constitute risk factors for various psychological issues, such as depression, anxiety, personality disorders, and low self-esteem [36,37]. Clarifying their relationship can contribute to identifying high-risk populations and understanding the mechanisms underlying comorbidity.

### Study objectives and hypotheses

Network analysis has gained prominence in psychological research for its ability to map complex interrelationships among variables and identify central and bridge nodes [38], thereby overcoming limitations of traditional variable-centered methods. By constructing an adult attachment–self-identity network, it becomes possible to pinpoint core components and their interconnections, thus providing precise targets for intervention. Therefore, this study employs network analysis to construct a network model examining the relationships between adult attachment and identity status among university students. The following hypotheses are proposed: H1: adult attachment and identity status will collectively form a closely interconnected network structure. H2: the network model of adult attachment and identity will contain both central symptoms and bridge symptoms. H3: significant differences in network characteristics are expected to emerge between different genders.

## Methods

### Power considerations

In the present study, we employed the Monte Carlo simulation approach—a general method for determining sample size in cross-sectional network analysis—using the R package powerly developed by Constantin et al. [39]. This method is widely adopted in psychometric network research [39–44]. It works by repeatedly drawing random samples from a population with a known network structure, thereby allowing researchers to evaluate the accuracy and stability of estimated network parameters under a given sample size [39]. As this is the first application of the method to examine the network linking adult attachment and self-identity, conducting this power analysis helps to verify whether our current sample (N = 624) provides sufficient reliability for detecting significant associations in the network, thereby strengthening the robustness of the study’s conclusions. Drawing on prior empirical work, we specified eight network nodes, An a priori power of 0.8, a density of 0.40, and a sensitivity of 0.60 [39]. Simulation results indicated that a sample size of 435 (95% CI) was required to achieve the pre-defined accuracy and power thresholds.

### Participants

Participants were recruited through convenience sampling from universities in Anhui, Shandong, and Jiangsu provinces between March 5 and March 15, 2025. A total of 778 questionnaires were distributed, with 624 returned, resulting in an effective response rate of 80.21%. The final sample consisted of 242 males and 382 females, aged from 17 to 23 years (M = 20.26, SD = 0.96). The study protocol was approved by the Ethics Committee of Huaibei Normal University (Approval No. HS45001), and all participants provided written informed consent.

### Measures

**Fig 1:**
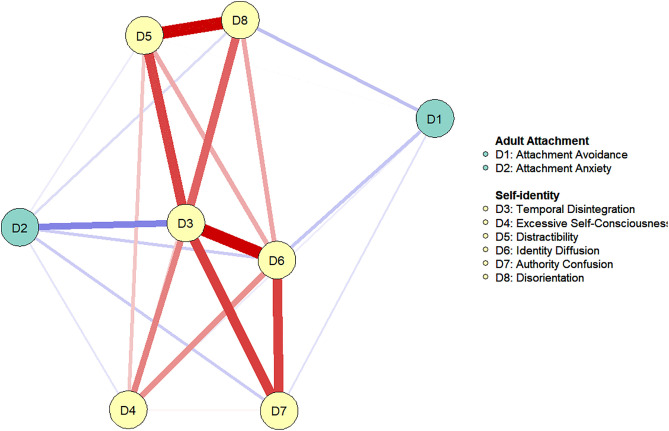
Adult attachment-self-identity network structure.

### Experience in close relationship inventory

The Experiences in Close Relationship Inventory [45], translated into Chinese by [46], comprises 36 items rated on a 7-point Likert scale (1 = strongly disagree, 7 = strongly agree). Eighteen items measure attachment avoidance, while the remaining 18 assess attachment anxiety. Items 3, 15, 18, 22, 25, 27, 29, 31, 33, and 35 require reverse scoring. Cronbach’s α was 0.847 for the full scale, with subscale reliabilities of 0.844 (avoidance) and 0.889 (anxiety).

### Self-identity questionnaire

Self-identity was assessed using the Self-Identity Questionnaire [34]. This 36-item scale measures six dimensions: temporal disintegration, excessive self-consciousness, distractibility, identity diffusion, authority confusion, disorientation. The development of this scale was grounded in Erikson’s theory of psychosocial development and fully incorporated the psychological characteristics of adolescents within the Chinese cultural context, making it a standardized instrument widely validated in domestic research on self-identity [47]. It utilizes a 5-point Likert scale, ranging from “strongly disagree” (1 point) to “strongly agree” (5 points) [47,48]. Reverse-scored items (e.g., items 5, 25, and 26) were recoded prior to analysis. The scale demonstrated good reliability, with a Cronbach’s α coefficient of 0.899.”

### Statistical analyses

Descriptive statistics were analyzed using SPSS 26.0. Network analyses were performed in R 4.4.2. A Gaussian Graphical Model (GGM) was employed on the full sample to construct a partial correlation network. Model selection was conducted using the Extended Bayesian Information Criterion (EBIC) in combination with the Least Absolute Shrinkage and Selection Operator (LASSO) to identify the optimal and most robust model among sparse network candidates generated under different LASSO regularization levels.

Node centrality was computed with the centralityPlot function to evaluate the overall importance of nodes, including strength, closeness, and expected influence. The correlation stability (CS) coefficient was calculated using the boot net package to assess the stability of the network and centrality indices; a CS value above 0.25 was considered acceptable. Bridge centrality metrics were computed using the network tools package. Finally, following previous studies [49,50], this study employed the Network Comparison Test via the R package to examine gender differences in networks from both global and local perspectives. Specifically, the network structure and global strength were used to assess global differences, while the edge strength was conducted to evaluate local differences. [51]. performing 5000 permutations at a significance level of‌‌ 0.05.

## Results

### Basic characteristics of test subjects

See Table 1 presents the basic statistical analysis results for the eight nodes in the adult attachment-self-identity symptom network, including the mean, standard deviation (*SD*), and predictability (*R*^2^) of each node.

**Table 1:** Basic information on nodes in the adult attachment-self-identity symptom network.

Label	Items	Mean ± SD	Predictability(*R*^2^)
D1	Attachment avoidance	3.85 ± 0.62	0.064
D2	Attachment anxiety	3.74 ± 0.96	0.213
D3	Temporal disintegration	3.32 ± 0.65	0.686
D4	Excessive self-consciousness	3.11 ± 0.58	0.278
D5	Distractibility	3.09 ± 0.54	0.495
D6	Identity diffusion	3.41 ± 0.65	0.586
D7	Authority confusion	3.64 ± 0.75	0.438
D8	Disorientation	3.21 ± 0.61	0.483

### Network analysis of adult attachment and self-identity among college students

The network structure of adult attachment and self-identity among college students is presented in Fig 1. The symptoms of self-identity form symptom clusters, while the attachment nodes are dispersed. The strongest association is between D5 (Distractibility) and D8 (Disorientation), followed by D3 (Temporal disintegration) and D6 (Identity diffusion), D3 (Temporal disintegration) and D7 (Authority confusion), D6 (Identity diffusion) and D7 (Authority confusion), and D3 (Temporal disintegration) and D5 (Distractibility). The strongest association is between D2 (Attachment anxiety) and D3 (Temporal disintegration), while D1 (Attachment avoidance) has the strongest associations with D6 (Identity diffusion) and D8 (Disorientation).

**Fig 2:**
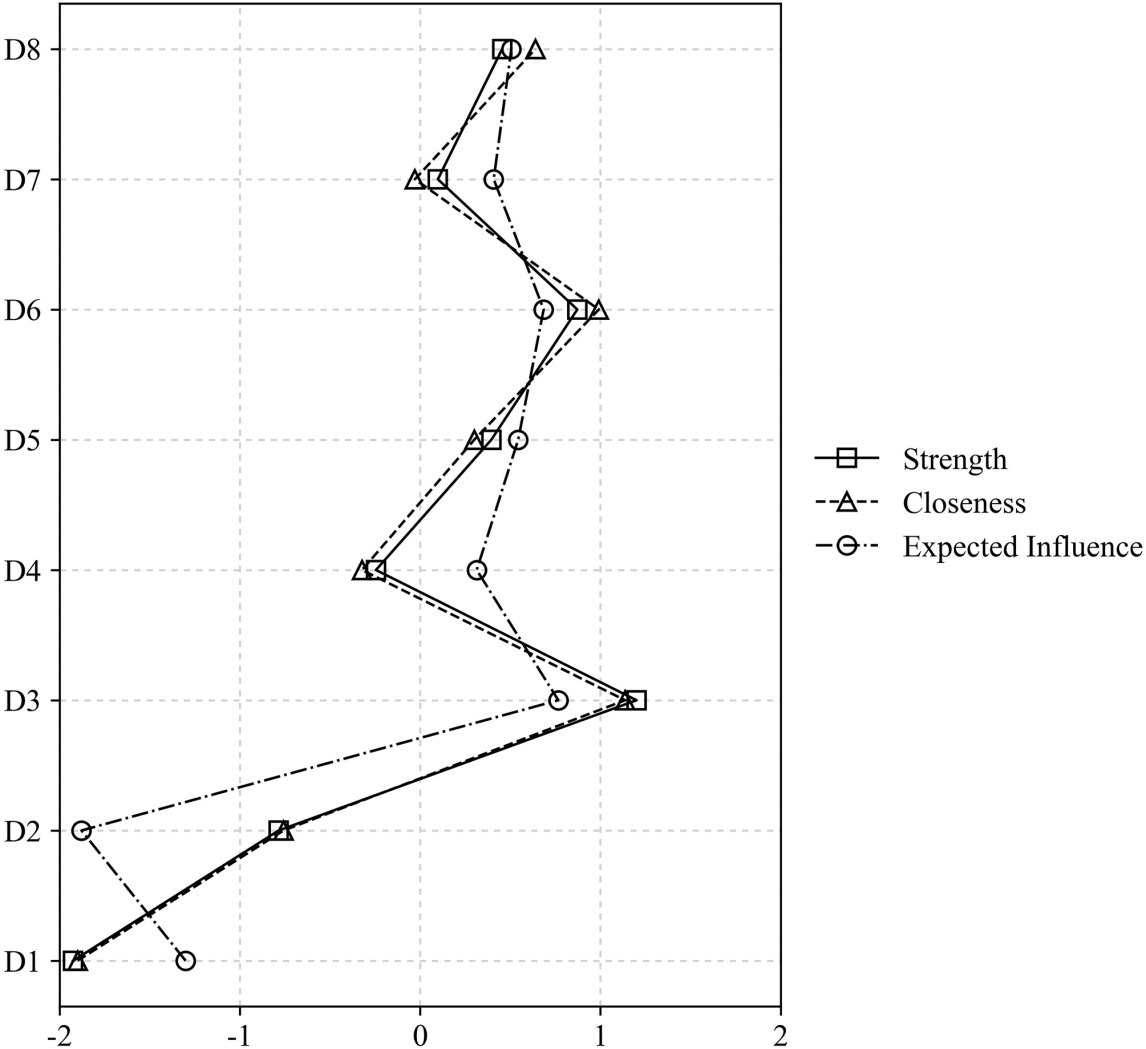
Centrality indices of nodes in the adult attachment-self-identity network.

Fig 2 and Table 2 shows the centrality indicators for each dimension of adult attachment and self-identity. D3 (Temporal disintegration) had the highest values for strength centrality, proximity centrality, and expected influence, with values of (1.197, 1.125, 0.751), indicating that temporal disintegration is a core manifestation of adult attachment and self-identity among university students. Next is D6 (Identity diffusion), with values of 0.864, 0.976, and 0.672 for strength centrality, proximity centrality, and expected influence, respectively. This indicates that temporal disintegration also plays an important role in adult attachment and self-identity among university students.

**Table 2:** Descriptive statistics of centrality indices.

Label	Items	Strength	Closeness	Expected Influence
D1	Attachment avoidance	−1.940	−1.899	−1.305
D2	Attachment anxiety	−0.795	−0.764	−1.867
D3	Temporal disintegration	1.197	1.125	0.751
D4	Excessive self-consciousness	−0.256	−0.330	0.314
D5	Distractibility	0.387	0.291	0.536
D6	Identity diffusion	0.864	0.976	0.672
D7	Authority confusion	0.091	−0.029	0.402
D8	Disorientation	0.451	0.631	0.497

### Network structure analysis of adult attachment and self-identity among college students of different genders

**Fig 3:**
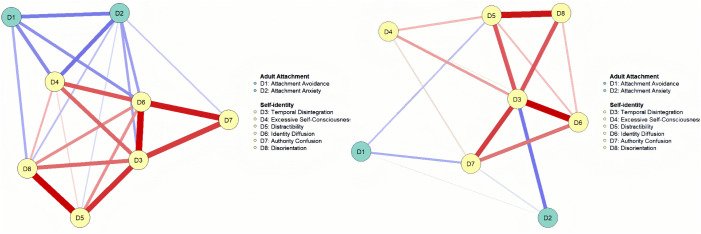
Network structure diagram of adult attachment and self-identity in female and male subgroups. **Note:** The left figure shows females; right figure shows‌‌ males.

**Fig 4:**
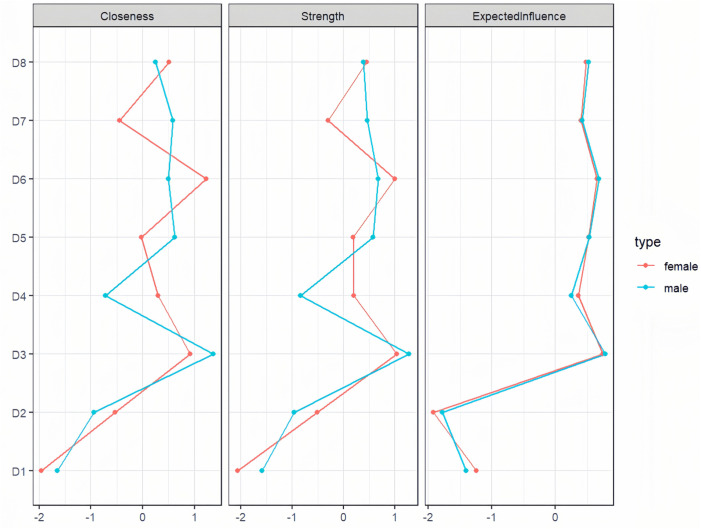
Adult attachment and self-identity network centrality indices for different ‌‌gender subgroups.

As shown in Fig 3 and Fig 4, the network structure of adult attachment–self-identity for different genders indicate that in both male and female networks, the node with the highest strength centrality is D3 (Temporal disintegration). In the male network, the node with the highest closeness centrality is D3 (Temporal disintegration), whereas in the female network, it is D6 (Identity diffusion). In both male and female networks, the node with the highest expected influence is D3 (Temporal disintegration).

The results of the network comparison between different genders show that (1) According to the network invariance test, there is no significant difference between the male and female groups (M = 0.148, P = 0.535). (2) According to the results of edge invariance test revealed that there were significant differences in the strength of some edges between males and females, as shown in Table 3, with the edge between D2 (Attachment anxiety) and D4 (Excessive self-consciousness) showing the most significant difference. (3) According to the results of the global strength invariance test, there was a significant difference between the male and female groups in terms of overall network connection strength (S = 0.614, P = 0.005). The global strength of females (3.231) was significantly higher than that of males (2.616). Although the overall structures of the two groups were similar, the global connection strength of the female network was significantly higher than that of the male network, indicating that the interconnections between variables in the female group were generally stronger and more tightly knit.

**Table 3:** The results of the network comparison.

Results of network comparison	*P* value	Statistic
**Male vs Female**	**Male vs Female**
Global strength invariance test	0.005	0.614（S）
Network invariance test	0.535	0.148 (M)
Edge invariance test		
D1-D2	0.027	0.126
D1-D4	0.030	0.103
D2-D4	0.013	0.148
D1-D5	0.050	0.065
D1-D6	0.091	0.115
D4-D6	0.061	0.147
D1-D7	0.041	0.107

Note: Only edges with statistically significant differences (*p* < 0.05) are shown.

### Analysis of bridge symptoms of attachment and self-identity among college students

**Fig 5:**
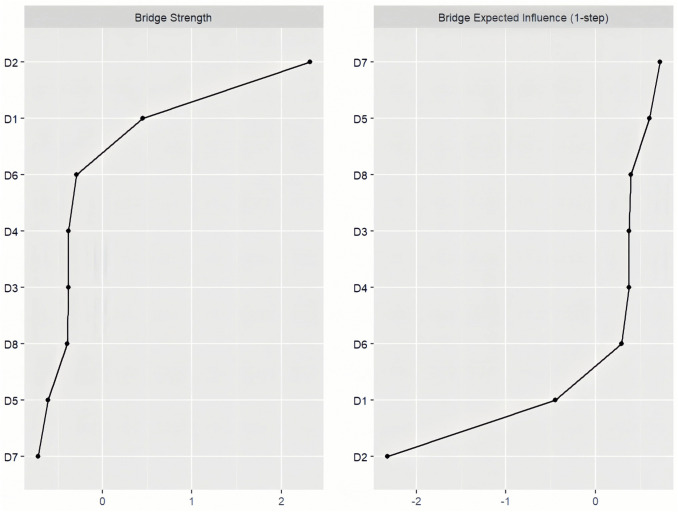
Bridge symptom analysis of adult attachment-self-identity network.

As shown in Fig 5, Bridge symptoms are those with the highest bridge centrality, representing the most influential symptoms within the network. Our analysis of bridge symptoms related to attachment-self-identity shows that D2 (Attachment anxiety) has the highest bridge strength, followed by D1 (Attachment avoidance). In terms of bridge expected influence, D7 (Authority confusion) has the highest bridge strength, followed by D5 (Distractibility), D8 (Disorientation), D3 (Temporal disintegration), and D4 (Excessive self-consciousness). These symptoms play a crucial role in connecting attachment and identity within the network.

### Stability and accuracy of network analysis

**Fig 6:**
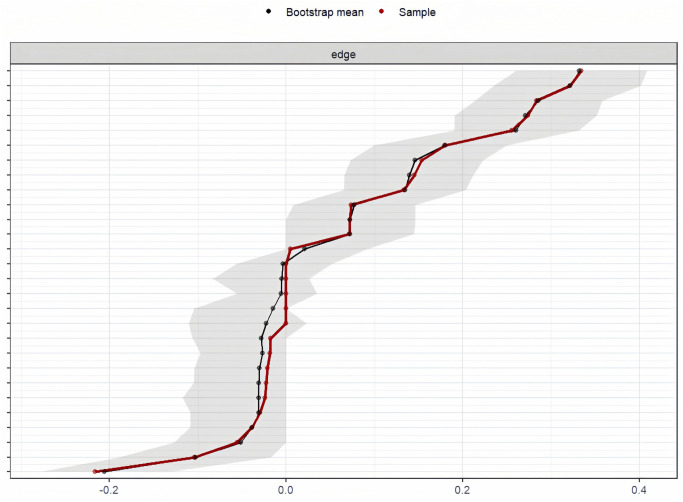
Accuracy analysis of edge weights in the adult attachment-self-identity network of college students. **Note:** The black line represents the average marginal weight of Bootstrap, and the red line represents the marginal weight in the research sample.

Stability tests were conducted on node centrality metrics and edge weights. The results showed that the CS values for the strength stability coefficient (CS), proximity centrality stability coefficient, and expected influence stability coefficient were all 0.75, indicating good stability. The Bootstrapped difference test results for edge weights are shown in Fig 6. The 95% confidence intervals for the sample set and the original data set overlap to a high degree, indicating that the edge weights of this network are relatively accurate.

## Discussion

### Core symptoms within the multidimensional integration theory of self-identity

The findings of this study reveal closely related symptom clusters within self-identity, with the closest association between D5 (Distractibility) and D8 (Disorientation), while D3 (Temporal disintegration), D6 (Identity diffusion) and D7 (authority confusion) are also closely related. These findings support the multidimensional integration theory of self-identity, which proposes that the construction and perception of personal identity are not unitary in nature, but rather consist of multiple interrelated dimensions. This is consistent with the theoretical proposition that self-identity constitutes an integrated multidimensional structure [34, 52], indicating that these dimensions are interwoven in individual experiences and collectively form the core characteristics of self-identity. Temporal disintegration emerged as the central node within the network structure, demonstrating the highest values in strength centrality, closeness centrality, and expected influence. This suggests that fragmentation or blurring of an individual’s sense of temporal continuity, goal-directedness, and future orientation may represent the most central issue in the self-identity development of college students, exerting the strongest predictive power and influence over other identity dimensions, such as identity diffusion, distractibility, and authority confusion. According to McAdams’ narrative identity theory, self-identity is essentially an integrative configuration achieved primarily through two pathways: synchronic integration, which coordinates the relationships among different roles an individual assumes within the same period, and diachronic integration, which connects self-images and experiences across different life stages [53]. Temporal dissociation directly undermines the temporal continuity required for diachronic integration, thereby impairing the individual’s ability to organize self-experience into a coherent “past–present–future” narrative. This disruption hinders the formulation of answers to “who I am” and “where I am going,” and complicates the integration of life events into a unified identity [53]. As noted by Zhao, a lack of temporal continuity can weaken an individual’s pursuit of future life goals [54]. Erikson’s concept of “psychosocial moratorium” also emphasizes that while exploring roles, adolescents must maintain an “inner continuity” to bridge childhood and adulthood [6]. Temporal dissociation precisely disrupts this continuity, leading identity exploration into confusion rather than constructive integration. Furthermore, D6 (Identity diffusion) was identified as another key node with high strength and betweenness centrality, underscoring the role of lacking a clear and stable self-definition in values, goals, and social roles within the self-identity network. Identity diffusion refers to an individual’s inability in synchronic integration, which involves reconciling the relationships between different roles assumed during the same period. These findings emphasize the importance of focusing specifically on experiences of temporal disintegration and identity diffusion in understanding and intervening in identity crises among college ‌‌students.

### The primary bridging symptom connecting adult attachment and self-identity networks

Further analysis indicates that, the bridge symptoms connecting adult attachment and self-identity include attachment anxiety, attachment avoidance, authority confusion, distractibility, disorientation, temporal disintegration, and excessive self- consciousness. Among these, D2 (attachment anxiety) demonstrated the highest bridge strength. The longitudinal study by Bourassa et al. indicated that hyperactivation strategies characteristic of attachment anxiety may lead to short-term psychological distress [55]. Notably, the strong association between attachment anxiety and temporal disintegration (D3) warrants further attention. According to Erikson’s theory, identity formation involves integrating diverse roles, competencies, dispositions, and social commitments into a coherent psychosocial structure that imbues life with unity and meaning [53]. This requires synthesizing self-representations across developmental stages—linking who one was, is, and aspires to be. Individuals high in attachment anxiety often struggle with this temporal integration, exhibiting difficulties in future planning, maintaining goal coherence, and sustaining a stable sense of self over time. Research indicates that insecure attachment impedes the development of a clear and stable self-concept [56]. This dynamic may be further amplified in China’s collectivist context, where self-construction is deeply embedded in social roles and relational obligations. Here, attachment anxiety not only affects internal emotion regulation but also shapes interpersonal strategies. Drawing on Goffman’s dramaturgical theory, social interaction can be understood as a performative act: individuals manage their public “front stage” presentation to uphold desired social identities while using the private “back stage” for reflection and emotional recalibration [57]. For those high in attachment anxiety, chronic sensitivity to social evaluation may lead to persistent hyper-vigilance on the front stage, manifested as excessive monitoring of social performance, anticipatory fear of rejection, and concerns about role inadequacy. This sustained focus on impression management can deplete cognitive resources and disrupt the temporal coherence of self-experience, thereby contributing to temporal disintegration.

On the other hand, D1 (Attachment avoidance) is another important bridge node, with the strongest connections to D6 (Identity diffusion) and D8 (Disorientation). Individuals with high attachment avoidance tend to suppress intimate needs and emotional expression, which may include expectations of others’ unreliability and an overemphasis on self-reliance. Individuals with high attachment avoidance scores often use suppression strategies that inhibit the processing of attachment-related information or events, which may lead to poorer emotional memory capacity [58]. This mechanism of processing emotional stimuli is also related to their lower emotional awareness. Avoidant attachment individuals attempt to prevent or inhibit any emotional states inconsistent with maintaining attachment needs or with their avoidance goals) [59]. Therefore, they exhibit a series of avoidance behaviors toward emotional stimuli. The long-term use of such avoidance strategies and independent problem-solving approaches leaves high-avoidance individuals lacking experience in establishing deep emotional connections. This may make their self-worth more susceptible to external evaluations. The combined effects of these factors may pose greater challenges for high-avoidance individuals in forming a stable self-identity and life direction, resulting in stronger identity diffusion and disorientation.

### Gender differences in network structures

Network structure invariance tests revealed no significant differences in the connectivity patterns between adult attachment and self-identity dimensions across male and female samples. This finding indicates that there are no significant gender differences in the core mechanisms linking attachment to self-identity, nor in the interrelationships between dimensions of self-identity. This aligns with the conclusions of previous research [28,30]. However, the edge invariance test revealed that there were significant differences in the strength of some edges between males and females (as shown in table 3), among which the edge between D2 and D4 showed the most significant difference. The test of global strength invariance revealed a significantly higher overall connectivity in the network of the female group compared to the male group. According to the network theory of psychopathology, this increase in global strength suggests that the activation of one symptom may more readily lead to the activation of other symptoms [44,60]. This finding has important implications, as research demonstrates that altering core nodes within a network can significantly impact the entire system [61]. When a node is activated, it rapidly propagates connections to other nodes, thereby activating the entire network [62]. Consequently, women’s self-identity development may be more susceptible to influence from attachment patterns, with a greater likelihood of mutual activation and reinforcement among network variables, potentially leading to reduced network stability. In contrast, the male group’s network exhibited looser connections, suggesting that the interplay between attachment patterns and self-identity may be relatively indirect or less pronounced. This finding also supports the research result that women tend to show higher attachment anxiety than men [27].

## Limitations

This study has several limitations. First, the representativeness of the sample is limited: participants were recruited exclusively from universities in Eastern China (specifically Anhui and Shandong provinces), with no inclusion of institutions from other regions such as Western or Northeastern China. Furthermore, convenience sampling was predominantly used, and the sample was largely drawn from teacher-training universities, resulting in insufficient representation of other types of higher education institutions, including comprehensive universities. In addition, the distribution of participants’ urban versus rural backgrounds was imbalanced (369 urban students vs. 255 rural students), potentially leading to an overrepresentation of students with urban household registration or those from areas with relatively greater educational resources. As a result, the current findings may be more applicable to populations similar to university students from educationally advanced regions in China. Future research should employ stratified sampling or comparable methods to broaden the sample to encompass individuals from diverse geographical regions, varied urban–rural backgrounds, different university types, and heterogeneous socioeconomic statuses. This would allow for testing the cross-regional robustness of the network structure and further exploration of how cultural or environmental factors may moderate the observed relationships.

Second, the network analysis can only identify associations among variables rather than elucidate causal mechanisms. Future studies should employ longitudinal designs or experimental methodologies to validate the dynamic pathways through which attachment influence identity formation. Third, this study relied on self-reported questionnaire data, which may be subject to social desirability bias. Future investigations may utilize experimental methods or biomarkers to obtain more objective data. Finally, the present study was limited to a single cultural group—Chinese university students. Although this focus enables a detailed examination within a specific sociocultural context, it restricts the cross-cultural generalizability of the findings. Future work should extend to Western student populations to enhance the cross-cultural applicability and robustness of the‌‌ results.

## Supporting information

S1 Data Data and Code. (ZIP)
